# Weekly versus biweekly bortezomib given in patients with indolent non-Hodgkin lymphoma: A meta-analysis

**DOI:** 10.1371/journal.pone.0177950

**Published:** 2017-05-22

**Authors:** Ting Yuan, Feng Zhang, Qing-min Yao, Yan-xia Liu, Xiao-juan Zhu, Xin Wang

**Affiliations:** Department of Hematology, Shandong Provincial Hospital affiliated to Shandong University, Jinan, Shandong, P.R. China; Mayo Clinic Rochester, UNITED STATES

## Abstract

**Background:**

Bortezomib is recently studied as a novel agent in indolent lymphoma. The optimal schedule of bortezomib used in indolent lymphoma is still uncertain.

**Methods:**

We did a systematic review and meta-analysis of the clinical trials comparing the efficacy and toxicity of the weekly and biweekly schedules of bortezomib in patients with indolent lymphoma. We searched Pubmed, Cochrane Library and Emabase from inception to July 29, 2016. The primary outcome was the overall response rate including the complete response rate and the partial response rate. The secondary outcomes were the proportions of patients in each group experiencing the adverse events including the neutropathy, fatigue, diarrhea, nausea and neutropenia.

**Findings:**

After final screening, six trials were considered eligible for analysis. The results showed that the overall response rate of biweekly schedule was higher than that of weekly schedule in indolent lymphoma (OR 1.691;95%CI 1.02–2.80). Furthermore, there were no significant differences between the two schedules of bortezomib for the main adverse events.

**Interpretation:**

The biweekly schedule of bortezomib was more effective than the weekly schedule in indolent lymphoma, with similar proportion of toxicities.

## Introduction

Patients with indolent non-Hodgkin lymphoma comprise approximately 25% of all new non-Hodgkin lymphoma cases. The indolent lymphoma, including follicular lymphoma, marginal zone lymphoma, and small lymphocytic lymphoma, typically follows a slow-growing course marked by frequent remissions to chemotherapy but inevitable relapses[1]. The therapeutic efficacy of indolent non-Hodgkin lymphoma has improved dramatically over the last decade as a result of the introduction of novel therapeutic agents[2–8]. For instance, the availability of new agents, including the anti-CD20 monoclonal antibody rituximab, has markedly improved outcomes in patients with follicular lymphoma[9].However, the disease is considered incurable and is characterized by repeated relapses after treatments[10].There is an ongoing need to develop alternate treatment approaches that may provide more effective and durable disease control[11].

Among the novel agents, bortezomib seems to play a pivotal role in the regulation of several cell signal pathways involved in the development of the lymphoma. Bortezomib inhibits the 26S proteasome reversibly, resulting in its antineoplastic effect through different mechanisms, such as inhibition of nuclear factor-κB, direct apoptosis, and antiangiogenesis effects[12]. Bortezomib has been successfully administered in combination with rituximab, bendamustine and additional chemotherapy combinations in the relapsed/refractory indolent lymphoma[13–15]. Several published randomized controlled trials have assessed the efficacy and toxicity of different modes of administration (weekly or biweekly). However, the optimal schedule of bortezomib remains to be investigated in indolent lymphoma. In this paper, we conducted a meta-analysis to evaluate the efficacy and safety of different administration schedules of bortezomib in indolent lymphoma.

## Methods

### Data sources

This meta-analysis was performed following the PRISMA protocol (S1 File). We did a systemic literature search for studies assessing the efficacy and safety of treatments for indolent lymphoma and the administration schedule of bortezomib, involving Pubmed, Cochrane Library databases and Embase. Search terms included “Follicular lymphoma”, “Marginal zone lymphoma”, “Small lymphocytic lymphoma” and “Bortezomib”. We searched all databases from inception to July 29, 2016. In addition, reference lists from the included studies were also manually searched.

### Study selection

To be eligible for inclusion, studies had to meet the following criteria. (1) Studies assessing the effectiveness and adverse effects of different schedules of bortezomib, alone or in combination with other treatment, for treating indolent lymphoma. (2) Randomized, controlled trials, retrospective or prospective cohort studies with a control (concurrent or historical) group. (3) The data from each treatment arm had to have been reported separately and had to be extractable. (4) The most detailed or the most recent article was chosen if data were presented in more than one article. (5) We limited our results to studies in English.

Studies those did not aim to evaluate the efficacy and safety of different schedules of bortezomib were excluded. Reviews, case reports, editorials or letters to the editor without original data were also excluded. A total of six studies were considered.

### Data extraction and assessment of study quality

Two authors independently reviewed the titles, abstracts and full texts of all studies in compliance with the inclusion and exclusion criteria. Disagreements were resolved by consensus.

The following information was retrieved from each study: first author’s name, publication year, study area, age of patients, sex, study design, study arms, number of total cases and number of patients having response in each arms, number of patients with neuropathy, fatigue, diarrhea, nausea and neutropenia in each arms, number of patients with grade 3 or higher neuropathy, fatigue, diarrhea, nausea and neutropenia in each arms.

### Quality assessment

The quality of each randomized controlled trials included in our meta-analysis was assessed according to the Jadad composite scale[16], a validated instrument that assesses generation of random sequences, randomization concealment, blinding, and study withdrawals to assign seven possible points reflecting the overall quality of clinical reports[16]. The quality scale ranged from 0 to 7 points, with a low-quality report receiving a score of 3 or less and a high-quality report receiving a score of at least 4. The quality of the non-randomized controlled trial was assessed using Newcastle–Ottawa quality assessment scale (NOS)[17]. The NOS evaluated three aspects including the selection of the study groups, the comparability of the groups, and ascertainment of either the exposure or outcome of interest for case-control or cohort studies respectively. The scores ranged from 0 to 9 points, with ≥5 points denoting high quality.

### Outcome measures

The primary outcome of the analyses was to compare the response rate of weekly and biweekly schedule of bortezomib administration in the treatment of indolent lymphoma. The secondary outcomes were to compare the proportions of the adverse events.

### Statistical analysis

Our meta-analysis was performed using Stata11. We inspected heterogeneity by chi-square test, if there was no significant heterogeneity between the results or heterogeneity is smaller (P ≥ 0.1,I ^2^≤ 50%), the fixed-effects model was used, for results showing high heterogeneity (P<0.1,I ^2^ >50%), we conducted the random-effects model. The odds ratio (OR) was applied as a surrogate for effect size according to heterogeneity. In the Forest plots, OR values >1 represented a direct association and <1 represented an inverse association. We performed sensitivity analysis by omitting studies included one by one. The likely presence of publication bias was examined by Begg Funnel plot. Except the heterogeneity, statistical significance was defined as P<0.05.

## Results

### Systematic literature review

**Fig 1:**
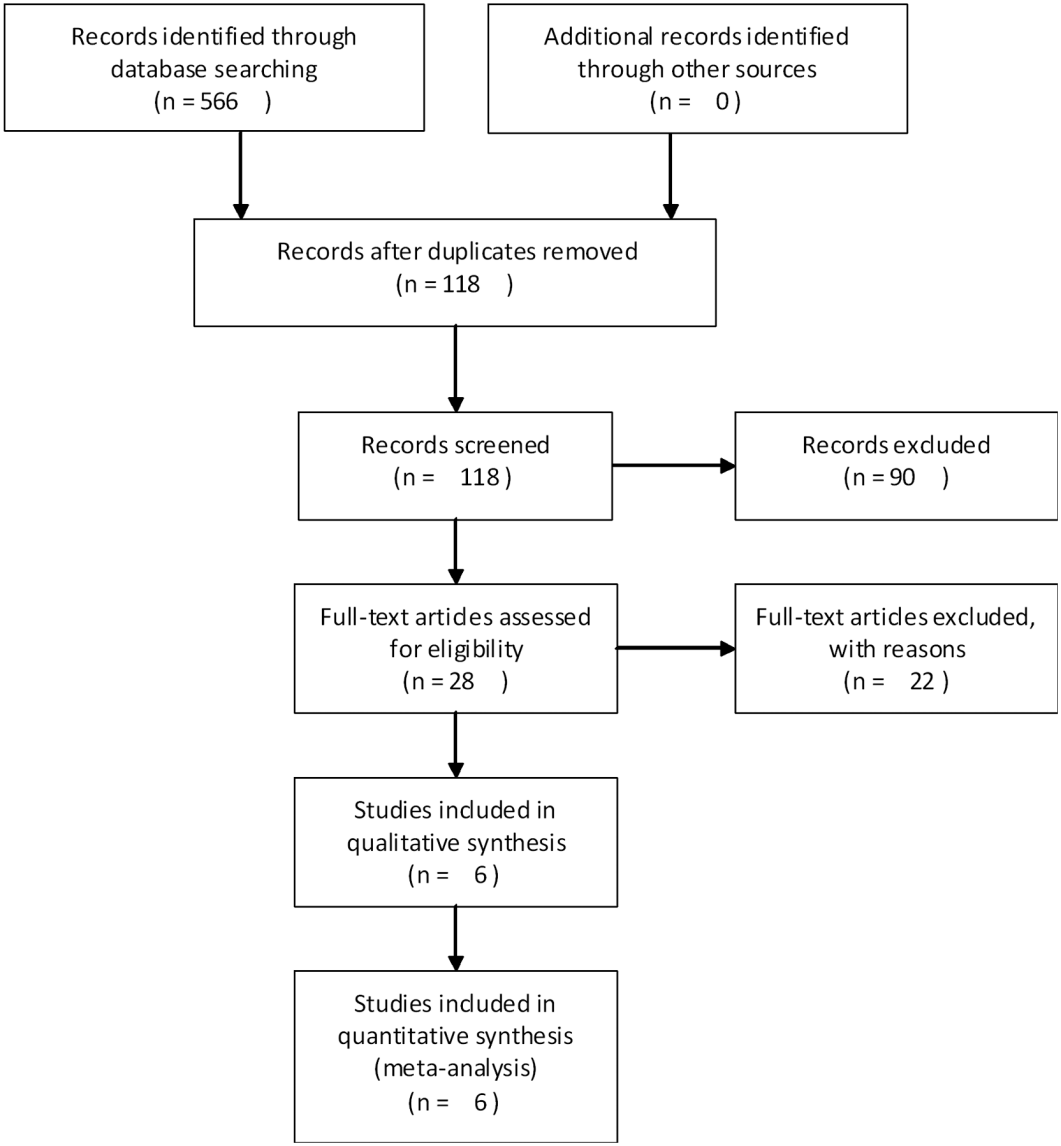
Flow diagram of the study selection process.

A total of 566 papers were selected through the electronic database searches with the specified terms. After initial review, 118 articles were remained after deleting any duplicates. Based on the titles and abstracts, 28 articles were selected for full-text review. After final screening, six records were considered appropriate for the current analysis. The flow diagram of literature search methodologies and included studies is presented in Fig 1.

### Study characteristics

The characteristics of the included six studies were shown in Table 1. The median age ranged from 63 to 65 years. Five of the included studies were phase II clinical trials[18–22], and one was phase I clinical trial[23]. Five trials were carried out in the United States[19–23], one was practiced in France[18]. The dosing, number of cycles, and synergistic chemotherapy regimens varied across studies. Three trials reported therapy with bortezomib alone[18,20,21], two trial reported therapy with bortezomib plus rituximab[19,22], and one trial reported therapy with bortezomib plus rituximab, cyclophosphamide and prednisone[23]. Table 2 shows the combined therapy and outcomes of studies included in our meta-analysis.

**Table 1:** The characteristics of 6 trials.

Study	Median age(M/F)(Y)	Sex(M/F)	Study Arm		Publication Year	Area	Study Design	Quality Score
			Weekly schedule	Biweekly schedule				
Ribrag V[18]	65	51/36	Bortezomib iv on days 1,8,15,22 of a 35-day cycle	Bortezomib iv on days 1,4,8,11 of a 21-day cycle	2012	FRA	Randomized, phase II trial	3(Jadad)
DeVos S[19]	64.5/63	41/40	Bortezomib iv on days 1,8,15,22 of a 35-day cycle	Bortezomib iv on days 1,4,8,11 of a 21-day cycle	2009	USA	Randomized, phase II trial	4(Jadad)
Gerecitano J[20]+O'Connor OA[21]	65.5+63	30/22	Bortezomib iv on days 1,8,15,22 of a 35-day cycle	Bortezomib iv on days 1,4,8,11 of a 21-day cycle	2009+2005	USA	Cohort, phase II trial	7(NOS)
Gerecitano J[23]	64	25/30	Bortezomib iv on days 2,8of a 21-day cycle	Bortezomib iv on days 2,5,9,12 of a 21-day cycle	2011	USA	Cohort, phase I trial	8(NOS)
Agathocleous A[22]	60/61	30/12	Bortezomib iv on days 1,8,15,22 of a 35-day cycle	Bortezomib iv on days 1,4,8,11of a 21-day cycle	2010	USA	Randomized, phase II trial	3(Jadad)

Abbreviations: M, male; F, female; Y, year; FRA, France; USA, United States; Jadad, Jadad composite scale; NOS, Newcastle–Ottawa quality assessment scale.

**Table 2:** Combined therapy and outcomes of studies included in the meta-analysis.

Study	Combined therapy	ORR		PFS		DOR		OS	
		w	b	w	b	w	b	w	b
Ribrga V[18]	none	22%	30%	6m	7m	15m	16m	Not reached	
DeVos S[19]	R	43%	49%	10m	5m	9.3m	Not reached	Not stated	
Gerecitano J[20]+O’Connor OA[21]	none	14%	64%	6.7m	4.8m	Not stated		Not stated	
Gerecitano J[23]	RCP	46%	64%	Not stated		Not stated		Not stated	
Agathocleous A[22]	R	67%	67%	Not stated		Not stated		Not stated	

Abbreviations: ORR, overall response rate; PFS, progression free survival; DOR, duration of response; OS, overall survival; w, weekly schedule; b, biweekly schedule; R, rituximab; C, cyclophosphamide; P, prednisone.

Of the six records, there were three phase II randomized controlled trials[18,19,22] and one phase I cohort trial[23]. The phase II trial conducted by Gerecitano J aimed to compare treatment schedules of weekly versus biweekly bortezomib in follicular lymphoma[20],it was an extension of a biweekly phase II trial of bortezomib in non-Hodgkin lymphoma, details of which had been presented by O'Connor OA[21], we extracted the data from these two phase II trials conducting by the same group in our meta-analysis.

### Response to treatment (6 trials, 280 patients)

**Fig 2:**
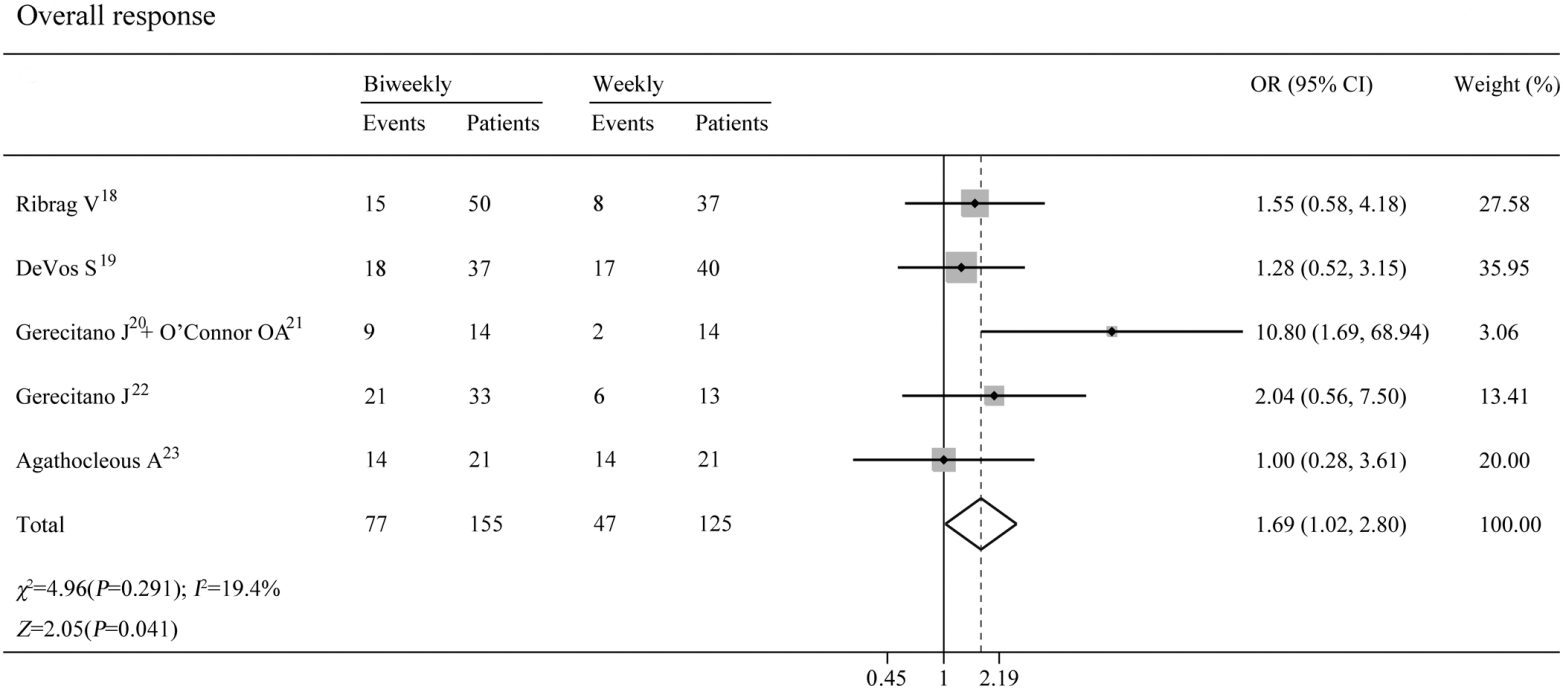
Pooled analyses of overall response.

In the six trials, overall response rates were evaluated by the International Working Group criteria. All studies were eligible for the analysis of overall response rates. There was no statistical heterogeneity between studies (P = 0.291;I^2^ = 19.4%). A fixed-effects model was used. The pooled sensitivity was 1.65(95% CI 0.98–2.76).(S1 Fig) Patients who underwent the biweekly schedule of bortezomib had a significantly higher overall response rate than those treated with the weekly schedule (P < 0.001)(OR 1.691; 95% CI 1.02–2.80).(Fig 2)

### Adverse events

**Fig 3:**
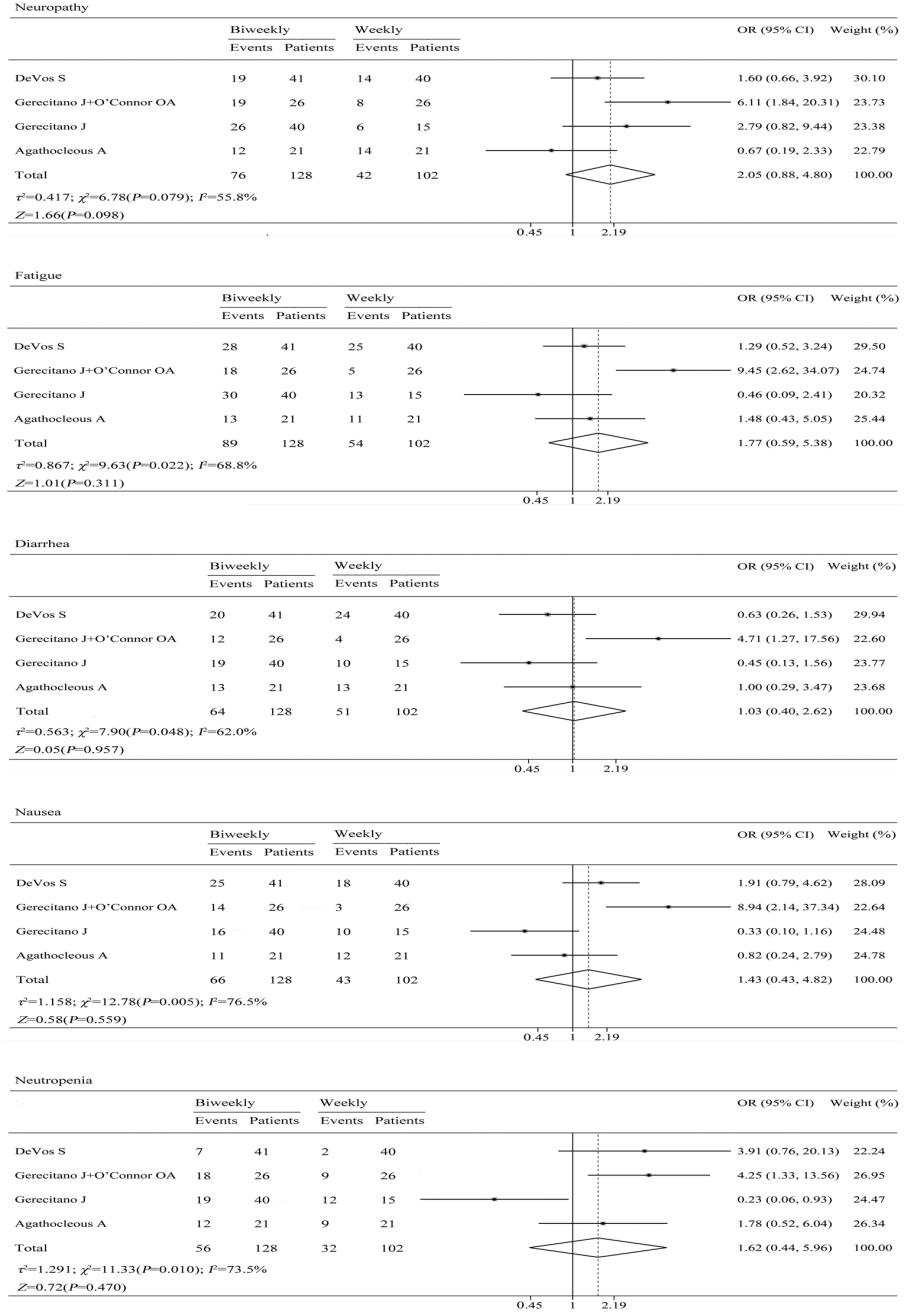
Pooled analyses of neuropathy, fatigue, diarrhea, nausea, and neutropenia.

**Fig 4:**
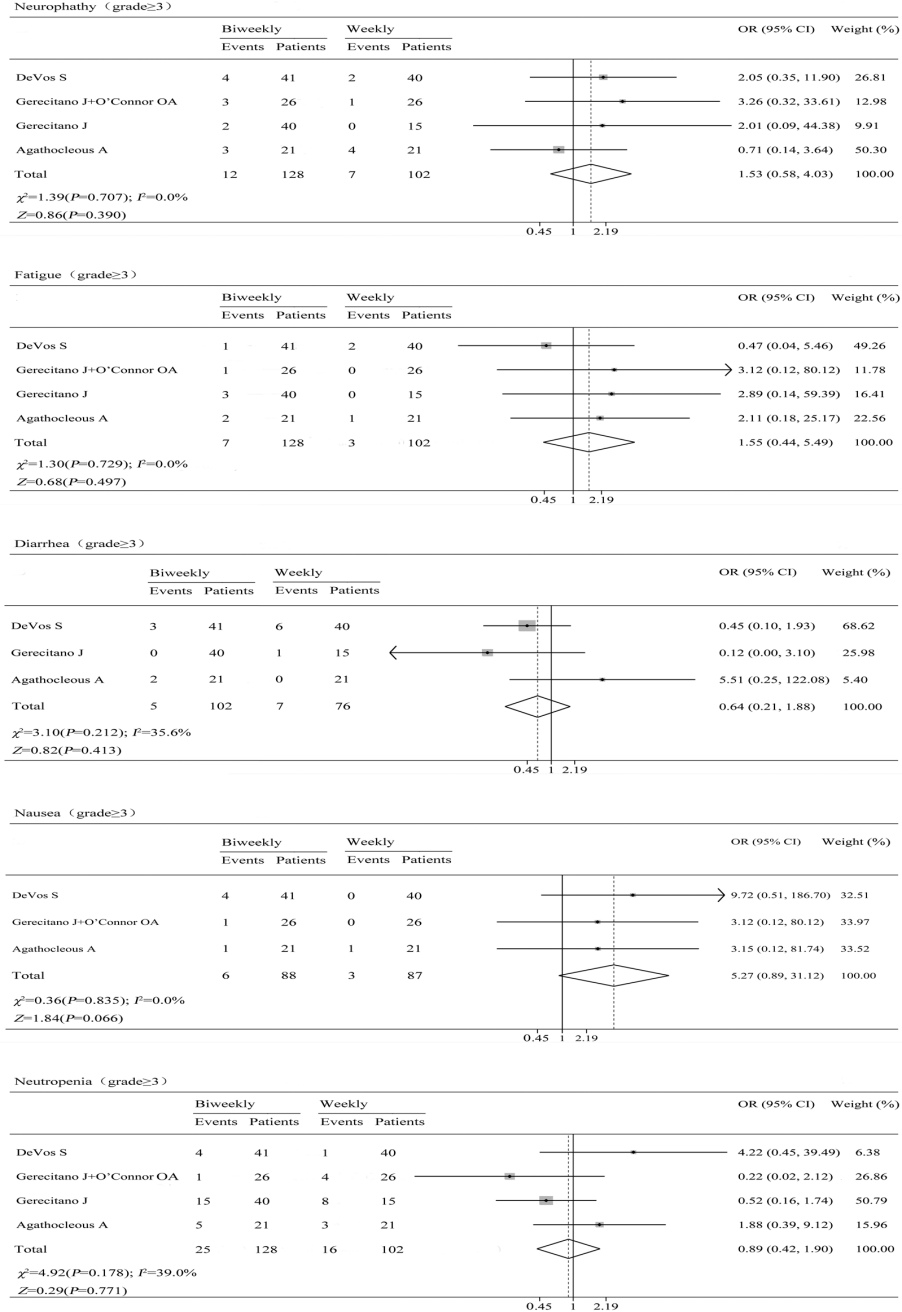
Pooled analyses of neuropathy, fatigue, diarrhea, nausea, and neutropenia (grade≥3).

We analyzed the common adverse events reported in the trials, the main adverse events included neuropathy, fatigue, diarrhea, nausea and neutropenia. The result indicated that there were no differences between the two schedules of bortezomib for the adverse events (Fig 3) and no differences were observed between arms for grade 3 or higher toxicities (Fig 4).

### Publication bias

Begg’test showed low risk of publication bias of studies.(S2 Fig)

## Discussion

Indolent lymphoma grows slowly but it is still an incurable disease. During the last few decades, great efforts have been made for the treatment of indolent lymphoma, but there still remains a number of challenges in the management. Patients with indolent lymphoma always receive multiple cytotoxic therapies during the course of the disease, and the multidrug resistance occurs frequently because of the repeated chemotherapy.

Bortezomib is the first proteasome inhibitor approved by the US Food and Drug Administration for using in multiple myeloma and relapsed/refractory mantle cell lymphoma. During the last few years, studies have indicated activity of bortezomib in indolent lymphoma whether botezomib is used as monotherapy or combined with other agents[24–39]. Goy A reported that bortezomib showed encouraging results in relapsed or refractory indolent lymphoma[24]. DiBella N demonstrated that the signal-agent bortezomib had modest activity against marginal zone and follicular lymphoma[26]. Jonathon B. Cohen indicated that the combination of bortezomib with R-CHOP was effective for indolent lymphoma[38]. Laurie H.Sehn confirmed that the addition of bortezomib to standard-dose R-CVP for advanced-stage follicular lymphoma was feasible and well tolerated with minimal additional toxicity[39].

Moreover, several phase I/II trials have demonstrated different response rates and toxicities with different bortezomib dosing schedules. DeVos showed that bortezomib administered with rituximab on a weekly schedule was as effective and less toxic than the same combination given on biweekly basis in patients with indolent lymphoma[19]. However, Ribrag V demonstrated that bortezomib given at biweekly schedule was more effective in patients with follicular lymphoma, no differences were observed between arms in the toxicities[18]

Bortezomib looks like a potential agent for the therapy of indolent lymphoma. Nevertheless, further studies are necessary to evaluate a proper schedule of bortezomib in indolent lymphoma. We performed a meta-analysis in an attempt to obtain further insight into the efficacy and safety of the two different schedules of bortezomib. This meta-analysis is the first study specifically addressing the comparison between the two schedules of bortezomib in indolent lymphoma.

Through a systemic literature search based on the exact inclusion and exclusion criteria, six clinical trials were included in our study. We judged the effectiveness of bortezomib by analyzing the OR of the overall response rate in all trials. The results showed that the biweekly schedule of bortezomib was more effective than the weekly schedule in indolent lymphoma (OR 1.691; 95% CI 1.02–2.80, P = 0.291;I ^2^ = 19.4%). We continued to compare treatment toxicities between the groups. The result showed that there were no differences between the two schedules of bortezomib for neuropathy, fatigue, diarrhea, nausea and neutropenia. Furthermore, no differences were observed between the arms for grade 3 or higher toxicities.

We noticed that the clinicians preferred the weekly schedule of bortezomib when treating the indolent lymphoma in combination with other drugs in the clinical trials[21,24,26,34]. Conversely, the biweekly schedule was common when bortezomib was used as monotherapy in indolent lymphoma[13,28–38]. The decisions of the schedule and dosage of bortezomib made by researchers depended on their experiences to a large extent. This meta-analysis aimed to provide evidence for the researchers to make proper clinical decisions and tried to eliminate the bias of personal experiences in the clinical trial designs, so that the patients with indolent lymphoma can achieve the best therapeutic effect.

There are several limitations of our study. Firstly, only three randomized controlled trials with a small number of patients were included in our study. Since bortezomib has not been included in the standard treatment for indolent lymphoma, the number of studies comparing the two schedules is small, which leads to a low level of evidence of the result. Secondly, since some studies did not show the exact data of the progression-free survival and the overall survival, we could not compare the survival outcomes of the two schedules which appeared more useful for the researchers to make treatment decisions. Thirdly, the treatment designs were not the same in all articles. The number of prior therapies, the clinical stage of the patients, the dosage and course of the bortezomib and the combined drugs were different across studies, which contributed to the heterogeneity. Finally, we could not avoid the measurement bias of the adverse effects because some studies did not list the data exactly, some trials included in our study compared the results in not only indolent lymphoma, but also some other types of lymphoma and the adverse events were showed in a summed table.

## Conclusion

This meta-analysis showed that the biweekly schedule of bortezomib was more effective than the weekly schedule in indolent lymphoma. The occurrence of adverse effects was similar. Long-term follow-up studies should be designed to help the clinicians to make more reasonable decisions.

## Supporting information

S1 File PRISMA 2009 checklist. (DOC)

S2 File PRISMA 2009 flow diagram. (DOC)

S3 File Search strategy for Pubmed database. (DOCX)

S1 Fig Pooled analysis of sensitivity. (TIF)

S2 Fig Begg’s funnel plot with pseudo 95% confidence limits. (TIF)
