# Supplementary material for: Weekly versus biweekly bortezomib given in patients with indolent non-Hodgkin lymphoma: A meta-analysis
Source: PLoS One. 2017 May 22;12(5):e0177950. doi: 10.1371/journal.pone.0177950 (PMC5439710; doi:10.1371/journal.pone.0177950)
Supplement: S2 File — (DOC) [file pone.0177950.s002.doc]

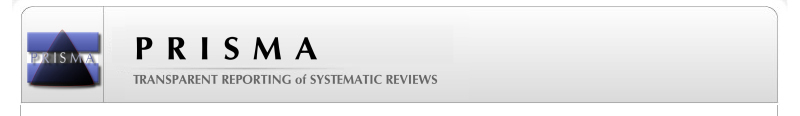
**PRISMA 2009 Flow Diagram**

**Screening**

**Included**

**Eligibility**

**Identification**

Records identified through database searching
(n = 566 )

Additional records identified through other sources
(n = 0 )

Records after duplicates removed
(n = 118 )

Records screened
(n = 118 )

Records excluded
(n = 90 )

Full-text articles assessed for eligibility
(n = 28 )

Full-text articles excluded, with reasons
(n = 22 )

Studies included in qualitative synthesis
(n = 6 )

Studies included in quantitative synthesis (meta-analysis)
(n = 6 )
