# Supplementary material for: Weekly versus biweekly bortezomib given in patients with indolent non-Hodgkin lymphoma: A meta-analysis
Source: PLoS One. 2017 May 22;12(5):e0177950. doi: 10.1371/journal.pone.0177950 (PMC5439710; doi:10.1371/journal.pone.0177950)
Supplement: S3 File — (DOCX) [file pone.0177950.s003.docx]

**Search strategy for Pubmed database**

#1. Follicular lymphoma

#2. Marginal zone lymphoma

#3. Small lymphocytic lymphoma

#4. bortezomib

#5. #1 OR #2 OR #3

#6. #4 AND #5
