# Supplementary figures and images for: Weekly versus biweekly bortezomib given in patients with indolent non-Hodgkin lymphoma: A meta-analysis
Source: PLoS One. 2017 May 22;12(5):e0177950. doi: 10.1371/journal.pone.0177950 (PMC5439710; doi:10.1371/journal.pone.0177950)

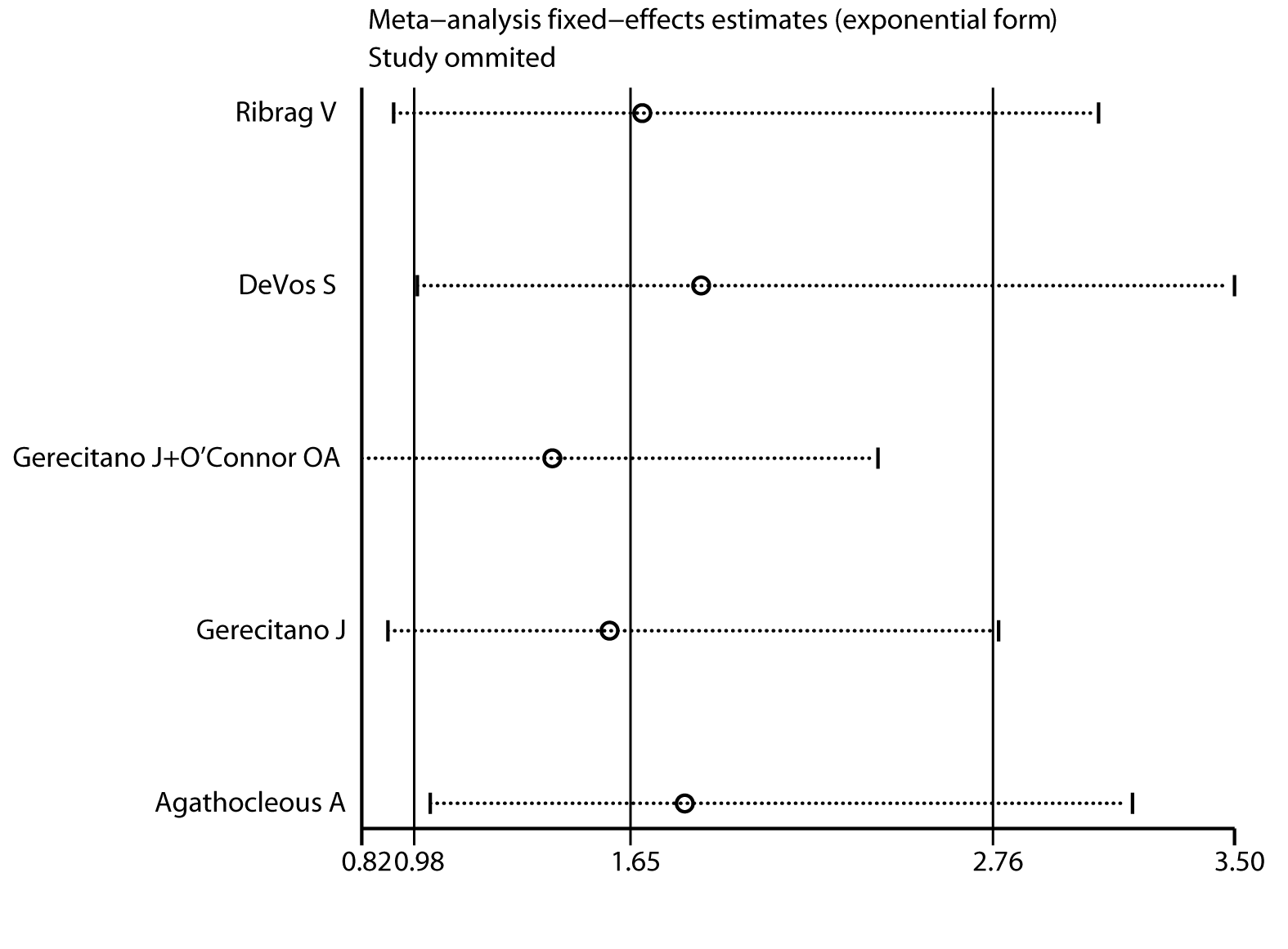

Supplement: S1 Fig — (TIF) [file pone.0177950.s004.tif]

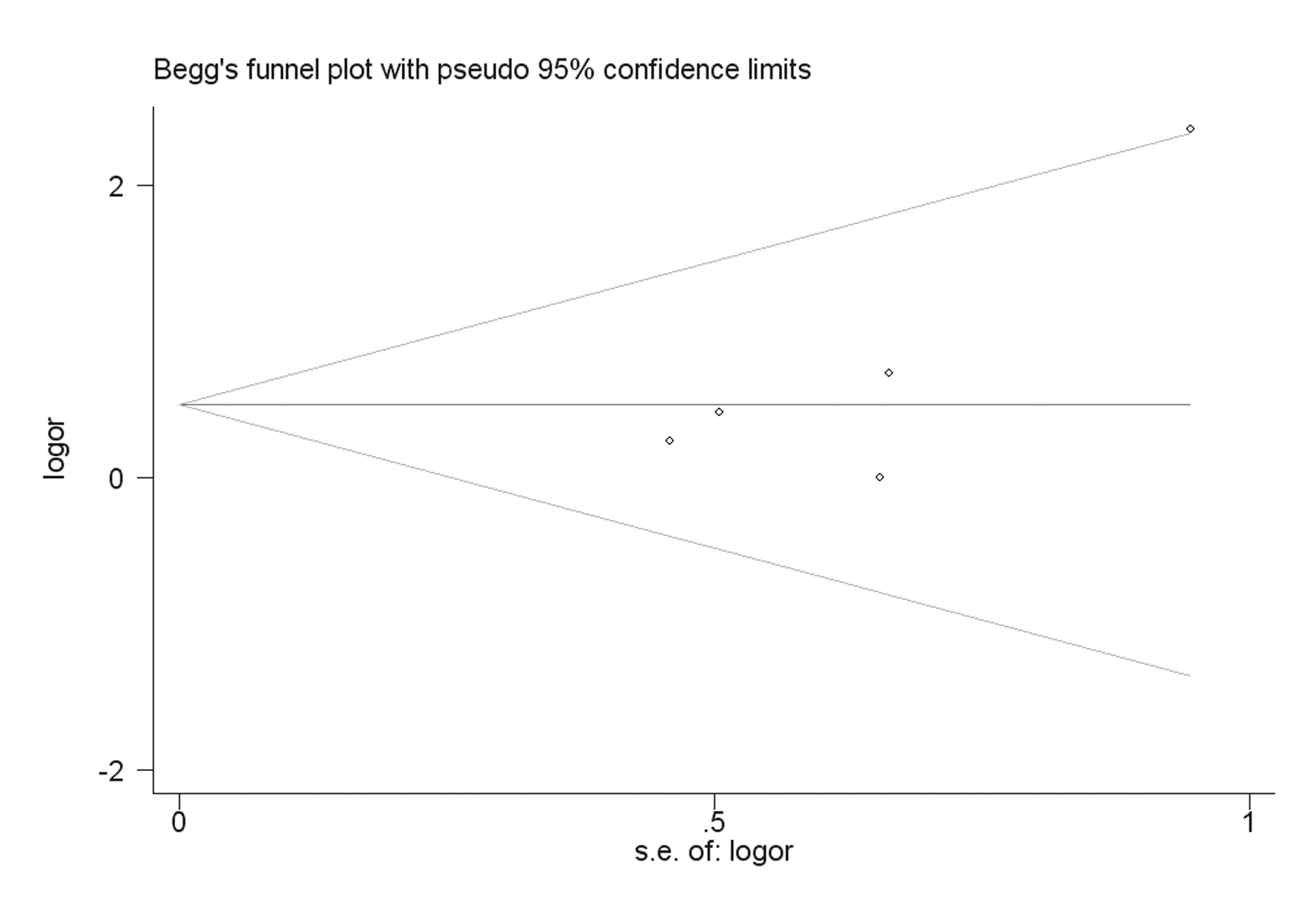

Supplement: S2 Fig — (TIF) [file pone.0177950.s005.tif]
